# Bariatric Surgery Induces Disruption in Inflammatory Signaling Pathways Mediated by Immune Cells in Adipose Tissue: A RNA-Seq Study

**DOI:** 10.1371/journal.pone.0125718

**Published:** 2015-05-04

**Authors:** Christine Poitou, Claire Perret, François Mathieu, Vinh Truong, Yuna Blum, Hervé Durand, Rohia Alili, Nadjim Chelghoum, Véronique Pelloux, Judith Aron-Wisnewsky, Adriana Torcivia, Jean-Luc Bouillot, Brian W. Parks, Ewa Ninio, Karine Clément, Laurence Tiret

**Affiliations:** 1 Institute of Cardiometabolism And Nutrition (ICAN), Assistance Publique-Hôpitaux de Paris, Pitié-Salpêtrière Hospital, Nutrition Department, F-75013, Paris, France; 2 Sorbonne Universités, University Pierre et Marie Curie (UPMC), Institut National de la Santé et de la Recherche Médicale (INSERM) UMR_S 1166, Nutriomics team, F-75005, Paris, France; 3 Sorbonne Universités, University Pierre et Marie Curie (UPMC), Institut National de la Santé et de la Recherche Médicale (INSERM) UMR_S 1166, Genomics and Pathophysiology of Cardiovascular Diseases team, F-75013, Paris, France; 4 Department of Medicine/Division of Cardiology, University of California Los Angeles, Los Angeles, California 90095, United States of America; 5 Sorbonne Universités, University Pierre et Marie Curie (UPMC), Post-Genomic Platform of Pitié-Salpêtrière (P3S), F-75013, Paris, France; 6 Assistance Publique-Hôpitaux de Paris, Department of Visceral Surgery, Pitié-Salpêtrière Hospital, F-75013, Paris, France; 7 Assistance Publique-Hôpitaux de Paris, Department of General, Digestive and Metabolic Surgery, Ambroise-Paré Hospital, F- 92100, Boulogne-Billancourt, France; Medical University Innsbruck, AUSTRIA

## Abstract

**Background:**

Bariatric surgery is associated to improvements in obesity-associated comorbidities thought to be mediated by a decrease of adipose inflammation. However, the molecular mechanisms behind these beneficial effects are poorly understood.

**Methodology/Principal Findings:**

We analyzed RNA-seq expression profiles in adipose tissue from 22 obese women before and 3 months after surgery. Of 15,972 detected genes, 1214 were differentially expressed after surgery at a 5% false discovery rate. Upregulated genes were mostly involved in the basal cellular machinery. Downregulated genes were enriched in metabolic functions of adipose tissue. At baseline, 26 modules of coexpressed genes were identified. The four most stable modules reflected the innate and adaptive immune responses of adipose tissue. A first module reflecting a non-specific signature of innate immune cells, mainly macrophages, was highly conserved after surgery with the exception of *DUSP2* and *CD300C*. A second module reflected the adaptive immune response elicited by T lymphocytes; after surgery, a disconnection was observed between genes involved in T-cell signaling and mediators of the signal transduction such as *CXCR1*, *CXCR2*, *GPR97*, *CCR7* and *IL7R*. A third module reflected neutrophil-mediated inflammation; after surgery, several genes were dissociated from the module, including *S100A8*, *S100A12*, *CD300E*, *VNN2*, *TUBB1* and *FAM65B*. We also identified a dense network of 19 genes involved in the interferon-signaling pathway which was strongly preserved after surgery, with the exception of *DDX60*, an antiviral factor involved in RIG-I-mediated interferon signaling. A similar loss of connection was observed in lean mice compared to their obese counterparts.

**Conclusions/Significance:**

These results suggest that improvements of the inflammatory state following surgery might be explained by a disruption of immuno-inflammatory cascades involving a few crucial molecules which could serve as potential therapeutic targets.

## Introduction

The widespread increase of obesity in the past few decades has become a major global health challenge [1]. A main burden of obesity lies in its interconnection with a number of metabolic and cardiovascular diseases including type 2 diabetes mellitus (T2D), dyslipidemia, hypertension and atherosclerosis.

Adipose tissue is increasingly recognized as a highly active organ involved in numerous metabolic, hormonal and immune processes, which plays a pivotal role in the development of obesity-related complications. A central mechanism linking obesity to metabolic dysfunctions is adipose inflammation which is caused by infiltration of immune cells into fat depots and increased production and secretion of pro- and anti-inflammatory factors into the circulation [2]. Besides macrophages which are the most abundant immune cells in fat depots, nearly all immune cell populations are modulated by the metabolic state of adipose tissue [3].

Bariatric surgery is the most effective therapy of severe forms of human obesity. It is associated with improvements in co-morbidities, including T2D, insulin resistance and cardiovascular risks [4, 5]. Several studies also reported changes in the inflammatory state both in adipose tissue and blood, particularly at early stage during the first year post-surgery [6]. However, the pathophysiological mechanisms behind these beneficial effects are poorly understood. Identifying the underlying molecular mechanisms that are critical to metabolic dysregulation and may contribute to surgery-induced improvements would be of considerable interest for targeting affected pathways.

Gene expression profiling has been instrumental in uncovering several biological pathways affected by weight loss. Transcriptomic studies of adipose tissue have highlighted major modifications induced by weight loss in lipid metabolism, extracellular matrix (ECM) organization and inflammatory processes [7–11]. However, all previous studies were based on the microarray technology which has been shown to be affected by several drawbacks. Recently the RNA-sequencing (RNA-seq) technology has emerged as a powerful and reliable tool for profiling gene expression which overcomes most limitations of microarrays [12].

In this study, we analyzed RNA-seq expression profiles in subcutaneous adipose tissue (SAT) from 22 obese women undergoing bariatric surgery. Expression levels were assessed before and 3 months after surgery. We investigated modifications in SAT transcriptome by applying both single-gene and network approaches. This combination of approaches provided insights not only into major changes in transcriptome profiles, but also into underlying molecular mechanisms causing disruption of biological pathways. Our study suggested that improvement of the inflammatory state after bariatric surgery might be explained by a break in the coexpression between inflammatory signaling pathways and a few crucial molecules involved in chemotaxis and activation of immune cells.

## Results

The mean age of the 22 women participating in the study was 37.5 ± 8.3 years. Bariatric surgery resulted in a decrease of the mean body mass index (BMI) from 46.5 to 39.9 kg/m^2^ and a significant improvement of all biological and metabolic parameters as expected (Table 1).

**Table 1:** Descriptive characteristics of the study population (*n* = 22 women).

	Before surgery	3 months after surgery	*P*-value
Age (years)	37.5 ± 8.3		
BMI (kg/m^2^)	46.5 ± 5.6	39.9 ± 4.9	<10^-4^
Type 2 Diabetes	3 (13.6%)	2 (9.1%)	NS
Metabolic syndrome	11 (50.0%)	8 (36.4%)	NS
Total cholesterol (mmol/L)	4.83 ± 0.70	4.44 ± 0.82	0.007
LDL-cholesterol (mmol/L)	3.14 ± 0.58	2.90 ± 0.70	0.07
HDL-cholesterol (mmol/L)	1.11 ± 0.32	1.03 ± 0.30	0.07
Triglycerides (mmol/L)	1.30 ± 0.59	1.13 ± 0.43	0.08
Glucose (mmol/L)	5.12 ± 0.65	4.81 ± 1.15	0.25
Insulin (μUI/ml)	21.6 ± 10.9	12.2 ± 6.73	2x10^-4^
CRP (mg/L)	9.36 ± 4.34	4.81 ± 2.43	0.005
IL-6 (pg/mL)	5.40 ± 3.11	4.06 ± 1.69	0.09
Leptin (ng/mL)	85.4 ± 35.5	39.6 ± 20.8	<10^-4^
Adiponectin (μg/mL)	4.59 ± 1.94	5.56 ± 3.04	0.051
Fat mass (kg)	61.9 ± 10.5	50.9 ± 8.3	<10^-4^
Lean mass (kg)	59.4 ± 6.4	53.5 ± 5.2	<10^-4^
Adipocyte volume (pL)	936.7 ± 188.4	823.1 ± 168.9	0.007
White blood cells (10^3^/mm^3^)	8.34 ± 2.26	6.38 ± 1.75	<10^-4^

Data are mean ± SD or n (%)

The mean number of read pairs produced by sequencing was 40.8 million pairs per sample (min: 35.5; max: 51.3). After mapping to the human reference transcriptome and filtering out genes not or weakly expressed (<10 counts in average at both times), 15,972 of the 23,329 annotated genes were included in analysis. In average, these 15,972 genes totalized 28.5 million read counts per sample (min: 23.8; max: 39.3).

### Effect of bariatric surgery on single gene expression levels

At a 5% false discovery rate (FDR), 1214 genes (7.6%) were differentially expressed (DE) three months after surgery (S1 Table). In the top list of upregulated expressions, there were several genes involved in mRNA processing and translation initiation such as heterogeneous nuclear ribonucleoproteins (HNRNP symbol), ribosomal proteins (RP symbol) and eukaryotic translation initiation factors (EIF and EEF symbols). The most significantly downregulated gene was *STMN2* which encodes stathmin-like 2, a protein primarily known for its role in dynamics of microtubules during neuronal growth [13] but also thought to be involved in the regulation of the fate of adipocyte differentiation [14].

Expression of the *LEP* gene encoding leptin, a key adipose-derived hormone whose serum level positively correlates to fat mass, was reduced by 31% (FDR = 0.001). *LOX*, a gene encoding lysyl-oxidase involved in collagen cross-linking was also downregulated (FDR = 5.7 10^-4^). By contrast, expression of the *ADIPOQ* gene encoding adiponectin, another adipokine inversely related to obesity-associated insulin resistance, did not significantly vary (FDR = 0.37). Expression of *FTO*, the most widely replicated gene in genome-wide association study of obesity, was not affected by surgery (FDR = 0.99). Several gene expressions that were reported in the literature to be modified by weight loss [7, 9–11] were found differentially expressed in the present dataset, including *ABCC6*, *ALDOC*, *B4GALT6*, *CCND2*, *CIDEA*, *EGFL6*, *ESR1*, *FADS1*, *FADS2*, *FASN*, *NQO1*, *SCD*, *SFRP4*, *SRPX* and *UCHL1*.

As the surgical procedure included 15 Roux-en-Y gastric bypass (RYGB) and 7 adjustable gastric banding (AGB), we tested whether differential expression was influenced by the type of procedure. No significant interaction between time and procedure was detected (all FDR > 0.24), even though there was a general trend towards larger differences in RYGB than in AGB. Results from the full negative binomial regression model including all covariates and interaction are shown in S2A Table and results for RYBG patients only are shown in S2B Table. Despite a smaller sample size, there were more genes that were differentially expressed in the RYGB group than in the whole population (1375 vs 1214 at 5% FDR).

### Gene Set Enrichment Analysis (GSEA) of genes differentially expressed by time

**Fig 1:**
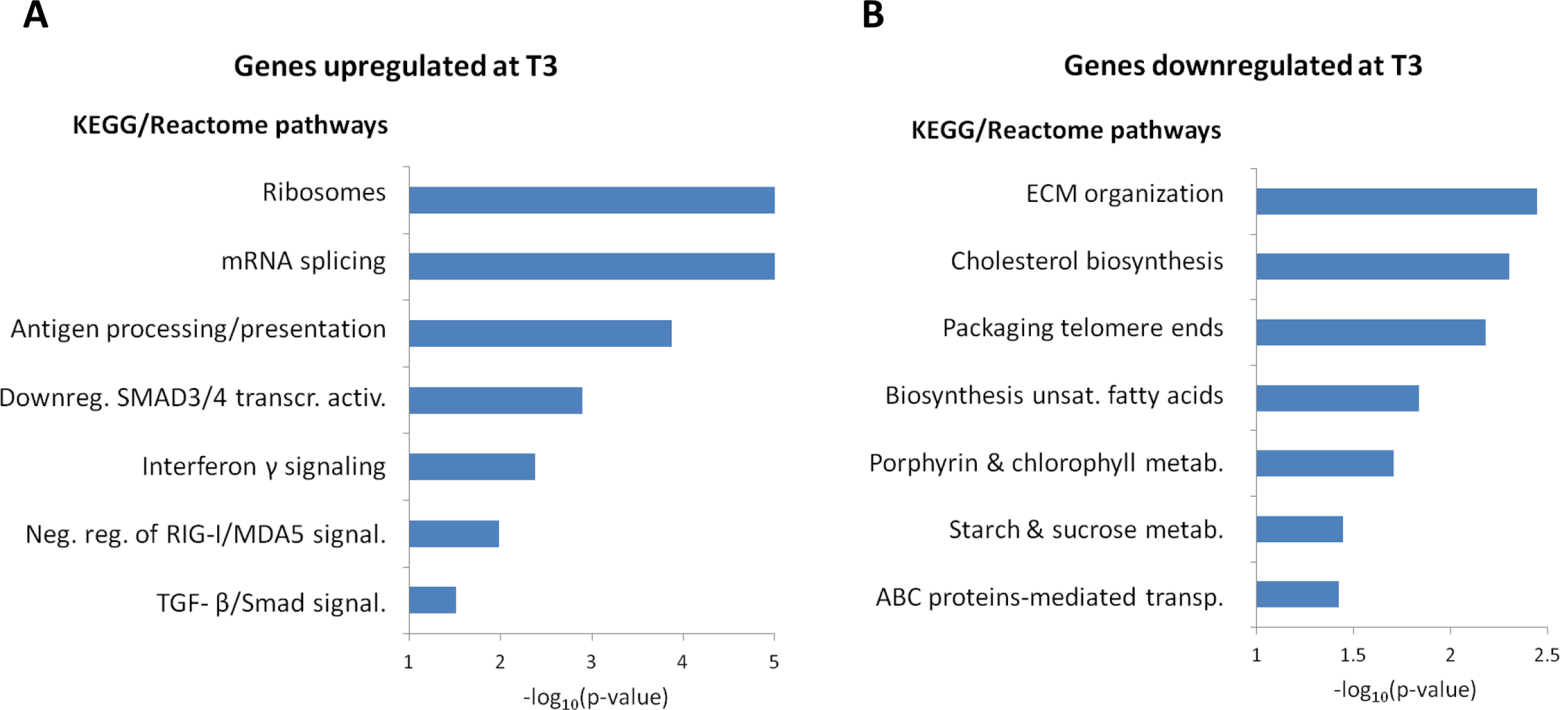
Pathways enriched in genes upregulated (A) and downregulated (B) after surgery-induced weight loss. The *x* axis indicates the significance of the GSEA score test for enrichment in the given pathway.

We applied GSEA [15] to explore functional pathways enriched among the most differentially expressed genes by time. For this analysis, genes were ranked according to their signed t-statistics obtained from the negative binomial model. Among genes upregulated at T3, 28 gene sets had a significant enrichment score at 5% FDR, 7 of them remaining after excluding overlapping gene sets (Fig 1A, S3 Table). The 2 most significantly enriched categories ("Ribosomes" and "mRNA splicing") reflected activation of the transcriptional and protein synthesis machineries. The category "Antigen processing/presentation" was driven by *HSPA1A* and *HSPA1B*, 2 members of the HSP70 protein family which mediate the folding of newly translated proteins, and *PSME1*, an interferon (IFN) γ-induced gene implicated in immune proteasome assembly and required for efficient antigen processing. The category "IFN-γ signaling" was driven by *PTPN2* and *IFNGR2* which both modulate IFN-γ signal transduction. These 2 categories were also enriched in genes of the class II/III major histocompatibility complex. The 3 remaining categories reflected downregulation of SMAD and MDA5 signaling pathways, and were all driven by *RPS27A*, a ribosomal gene encoding an ubiquitin gene [16] possibly involved in protein degradation.

When focusing on genes downregulated at T3, 11 gene sets had a significant enrichment score, 7 of them remaining after excluding overlapping gene sets (Fig 1B, S4 Table). A first category was composed of genes involved in ECM organization, including several collagen and metalloproteinase genes. Two categories included genes involved in the biosynthesis of cholesterol and unsaturated fatty acids, respectively, and one category corresponded to the ATP-binding cassette family of proteins involved in cholesterol transport. A category "Packaging telomere ends" included several histone genes (HIST symbol) as well as *TINF2* encoding shelterin, a protein involved in maintaining telomere integrity [17]. The two last categories concerned metabolism of porphyrin and chlorophyll, and metabolism of starch and sucrose, respectively.

A last analysis considering genes ranked according to their *absolute* t-statistics indicated 6 enriched gene sets, 2 of them being not detected by analyses separating up- and downregulated genes: one category included genes involved in bile acid metabolism (9 genes); the other category included genes involved in activation of the NF-κB pathway by TAK1 (8 genes, the top one being *IRAK2*) (S5 Table).

### Weighted gene coexpression network analysis (WGCNA) at baseline

To get deeper insight into the biology of SAT, we sought to identify networks of coexpressed genes by applying WGCNA [18] to the 10,000 most variable gene expressions at baseline. Genes not selected for this analysis, i.e. the 5972 least variable genes, were highly enriched in nuclear genes (P = 7.1 10^-50^).

**Fig 2:**
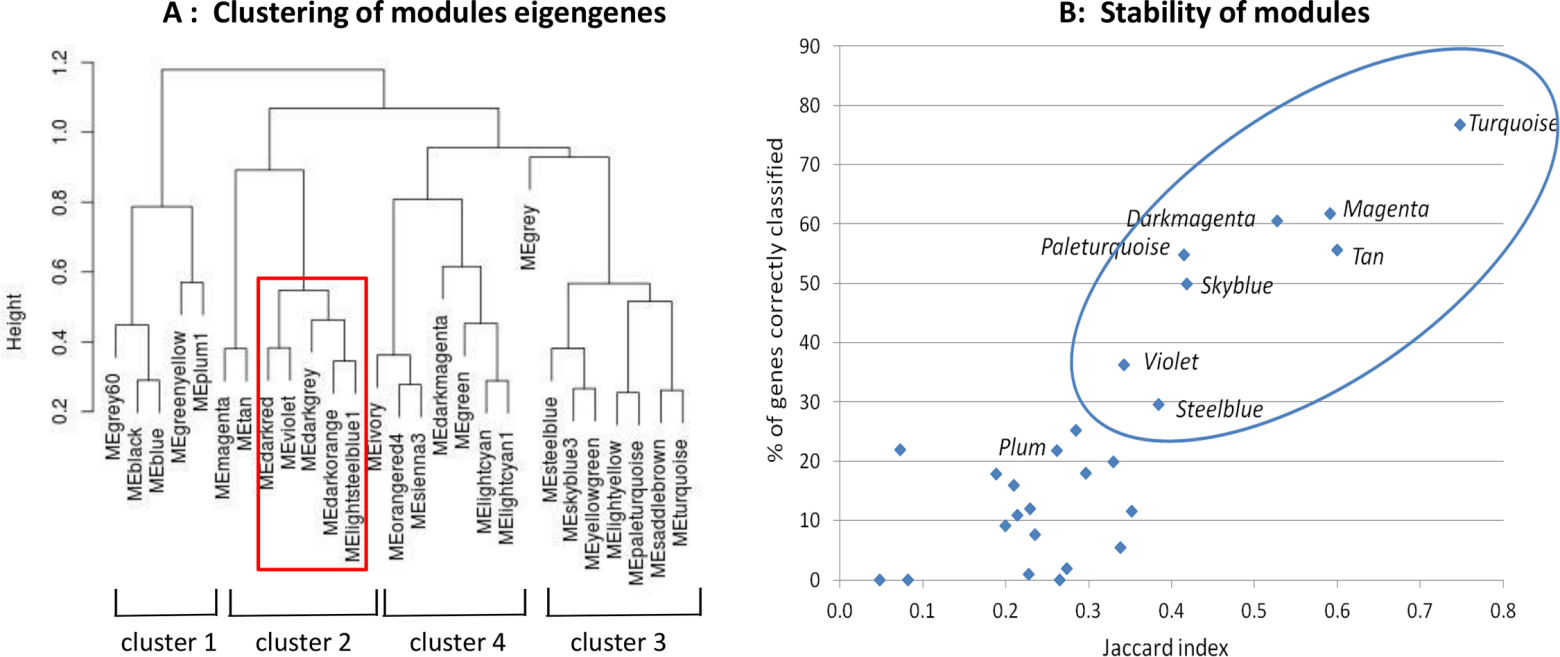
Gene coexpression networks at baseline identified by WGCNA. (A) Hierarchical clustering of module eigengenes. The open red square indicates the 5 modules with the highest proportions of differentially expressed genes (>20%). (B) Stability of modules by Jackknife analysis. The *x* axis indicates the mean Jaccard index and the *y* axis the proportion of genes correctly classified (i.e. attributed to the proper module in ≥ 18 out of 22 sub-analyses). The ellipse surrounds the most stable modules.

Twenty-seven gene coexpression modules were detected by WGCNA. The grey module comprising genes not being assigned to any module was not further considered. The 26 remaining modules, whose size varied from 56 to 1901 genes, could be regrouped into 4 large clusters (Fig 2A). The proportion of DE genes within each module varied from 0.5% to 28.4%. Interestingly, the 5 modules showing the highest proportions of DE genes (>20%) tended to cluster together (Fig 2A, red square).

**Fig 3:**
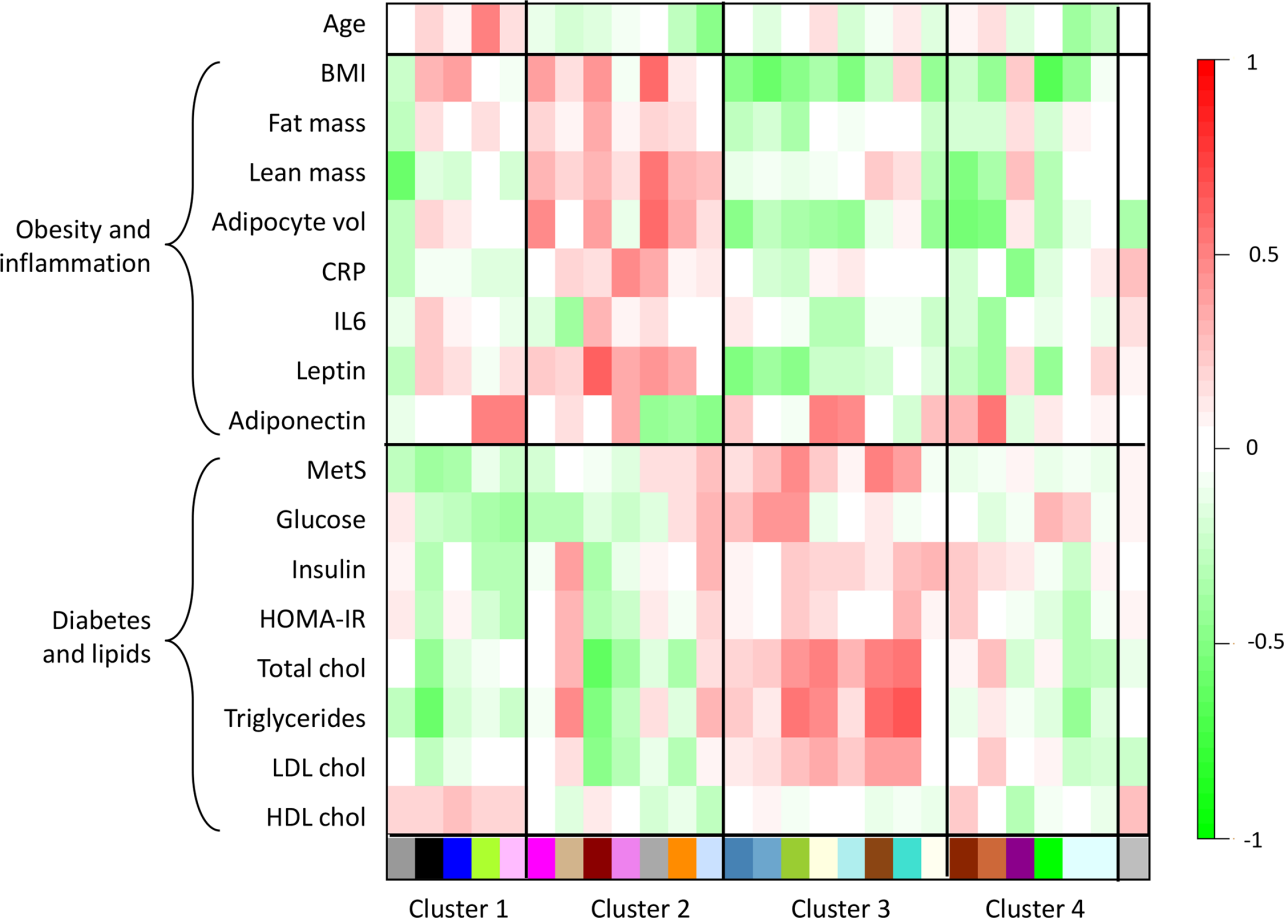
Correlations between module eigengenes and patient's characteristics at baseline. Clinical and biological traits (rows) were regrouped in 2 categories (obesity and inflammation-related traits, glucose and lipid homeostasis-related traits). Module eigengenes (columns) were clustered as in Fig 2. In the heatmap, red color represents positive correlation and green color negative correlation.

When examining the relationships between modules and phenotypic traits measured at baseline, a structure into 4 blocks reflecting the 4 aforementioned clusters was apparent (Fig 3). Clusters 2 and 3 had a mirror-like structure, cluster 2 being positively associated to obesity- and inflammation-related traits (BMI, fat mass, lean mass, adipocyte volume, C-reactive protein [CRP], interleukin[IL]-6, leptin and adiponectin) and negatively related to lipids and diabetes-related traits (total, LDL and HDL cholesterol, triglycerides, metabolic syndrome [MetS], glucose, insulin and Homeostasis Model Assessment of Insulin Resistance [HOMA-IR] index), while cluster 3 showed associations in the opposite direction. Clusters 1 and 4 showed a less clearcut structure. The complete lists of genes belonging to the different modules are provided in S6 Table.

The stability of modules was assessed by the Jackknife technique. Stability criteria that were used were the Jaccard index, the proportion of correctly classified genes and the mean eigengene correlation (see Methods). Worthy of note, modules including the highest proportion of DE genes showed a relatively low stability (S7 Table). For further biological interpretation, we focused on the most stable modules as shown in Fig 2B.

### Biological significance of RNA-seq modules at baseline

We examined modules according to several criteria: 1) the Gene Ontology (GO) functional categories significantly enriched; 2) the detailed exploration of the network formed by the top 30 hubgenes displayed as circle plot; and 3) the Spearman's rank correlation of the module eigengene with baseline phenotypic traits. For each module, gene module membership, stability score and genes belonging to enriched categories are given in S8 Table.

**Fig 4:**
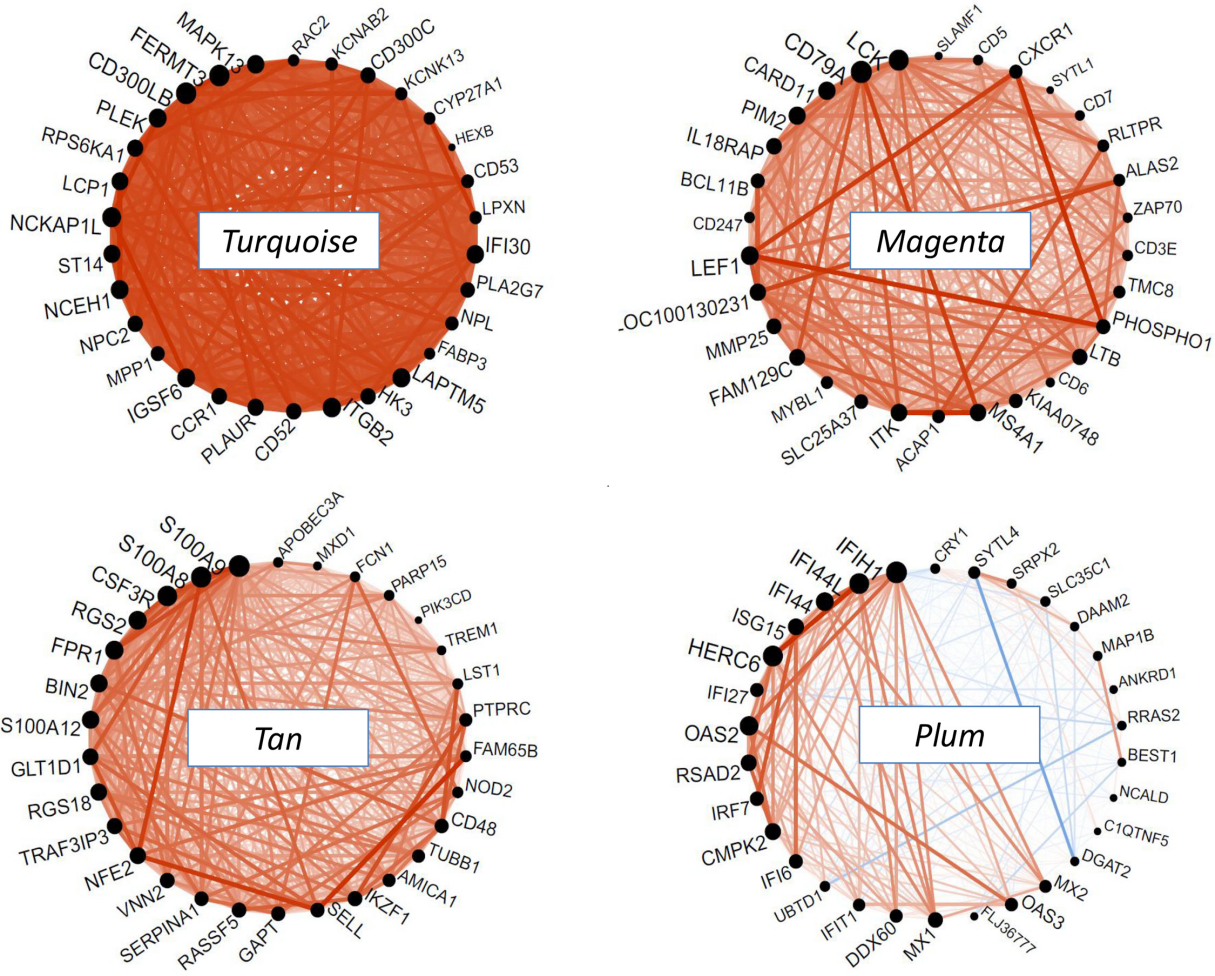
Network circle plots of immune-response modules at baseline. The figure depicts the connectivity patterns between the top 30 hubgenes of each module. Genes are ranked in the anticlockwise direction according to decreasing module membership. The size of each black circle indicates the gene connectivity (number of connected genes). An orange line indicates a positive correlation and a blue line a negative correlation between any 2 genes. The *turquoise* module reflects a general immune response, the *magenta* module reflects lymphocyte activation, the *tan* module reflects neutrophil activation and the *plum* module reflects the interferon signaling pathway.

The *turquoise* module was the largest module. It was enriched for the GO process "immune response" (FDR = 7.9 10^-16^) and was associated to a very dense network of hubgenes (Fig 4). The top hubgenes were *MAPK13*, a member of the MAP kinase signal transduction pathway, and *FERMT3*, which encodes a protein of the kindlin family involved in integrin activation and cell adhesion. This module included many inflammatory and immune proteins such as CD molecules (including *CD14* and *CD33*, 2 monocyte markers), chemokines and proteins of the complement system. This module may reflect the general obesity-associated immune response characterized by monocyte recruitment and macrophage infiltration. The eigengene positively correlated to lipid variables including total cholesterol (r = 0.53) and triglycerides (r = 0.68).

The *magenta* and *tan* modules were close to each other in the hierarchical clustering (Fig 2A). The *magenta* module was a highly connected module enriched for the GO category "cell surface receptor linked signal transduction" (FDR = 2 10^-9^) which comprised many genes coding for cytokine receptors as well as CD molecules. The top 30 hubgenes (Fig 4) were present in all sub-sample analyses, indicating a high stability of the network. Among them several genes were involved in the lymphocyte-activation pathway: *LCK* encoding the lymphocyte-specific protein tyrosine kinase, *CD79A* and *MS4A1* (alias *CD20*)—two B-lymphocyte antigens strongly linked one with each other, and *CARD11—*a molecule with a caspase domain essential for T-cell activation. The module also contained *CD8A* and *CD8B* characterizing the specific subpopulation of CD8^+^ T cells that play an essential role in the initiation and propagation of adipose inflammation in rodents [19]. The *magenta* module was additionally enriched for the GO class "hemoglobin complex" (P = 1.8 10^-6^). This enrichment was due to the inclusion of several hemoglobin genes (*HBA1*, *HBA2*, *HBB*, *HBD*, *HBM*, *HBQ1*, *HBG1*, *HBG2*) and erythrocyte-specific genes (*AHSP*, *ALAS2*, *SLC4A1*). The *magenta* module eigengene tended to be positively related to all obesity-related traits, although only the correlation with adipocyte volume reached significance (r = 0.49).

The *tan* module was enriched for the GO category "defense response" (FDR = 6.5 10^-11^) which included several receptors of the innate immune response such as chemokine receptors or Toll-like receptors. Several of these proteins act as monocyte chemoattractants. The dense network of the 30 hubgenes (Fig 4) was driven by 2 genes, *S100A8* and *S100A9* *(*calgranulin A and B, respectively), which form a heterodimer S100A8/A9 (or calprotectin) originally discovered as an immunogenic protein expressed and secreted by neutrophils [20]. The module also contained the gene encoding *S100A12* (calgranulin C). Calgranulins bind to pattern recognition receptors and serve as trigger factors of inflammation in concert with *IL1B*, another member of the module [21]. The 3^rd^ hubgene of the module was *CSF3R* which encodes the receptor for colony stimulating factor 3, the principal cytokine controlling neutrophil development and function [22]. The *tan* module might then reflect coexpression of genes characterizing the inflammation associated to neutrophil activation and monocyte infiltration of adipose tissue. As the *magenta* module, the *tan* module tended to be positively related to obesity-related traits, but in addition it significantly correlated to MetS-related traits, in particular triglycerides levels (r = 0.46).

**Fig 5:**
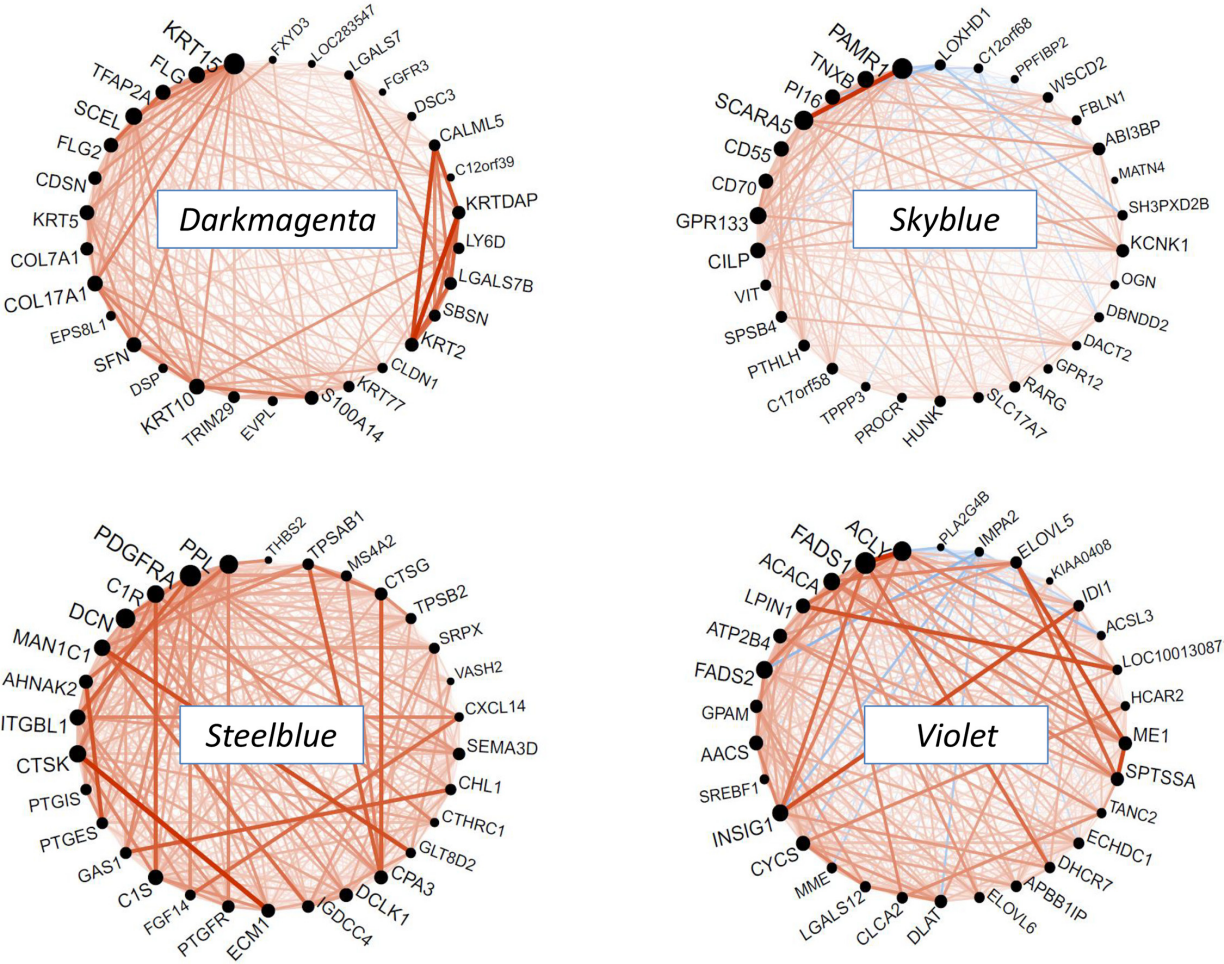
Network circle plots of other modules at baseline. See legend of Fig 4. The *darkmagenta* reflects ectoderm, the *skyblue* and *steelblue* modules reflect the extracellular matrix and the *violet* module reflect lipid metabolism.

The *darkmagenta* module was a small module strongly enriched in the GO biological process "ectoderm development" (FDR = 1.8 10^-19^). Several genes of this module coded for proteins involved in the differentiation of keratinocytes, such as keratins, intermediate filaments and proteins of the cornified envelope (Fig 5). The top hubgene *KRT15* has been implicated in breast and lung cancers [23, 24]. Among the major contributors of this module were also *FLG* coding for fillagrin, a protein that aggregates keratin intermediate filaments, and *TFAP2A*, a transcription factor expressed in the ectoderm. A small sub-network was formed by *KRT2*, *SBSN*, *LGALS7B*, *LY6D*, *KRTDAP* and *CALML5*, all genes coding for proteins involved in differentiation of keratinocytes. The *darkmagenta* module was negatively associated to CRP levels (r = -0.46).

The *paleturquoise* module was a large module moderately enriched for the GO biological process "transcription" (FDR = 2.5 10^-2^). Thirty-eight genes in the module were zinc finger proteins (ZNF symbol) which are major transcriptional regulators whose role in adipogenesis is increasingly recognized [25]. The top hubgene (data not shown) was *NIPBL* encoding delangin, a member of the cohesin complex which plays a key role in transcription [26]. Other hubgenes were *ARFGEF2*, an exchange factor involved in the trans-Golgi trafficking of proteins [27] and *NCOA2* (alias *TIF2*) which modulates energy metabolism [28]. The *paleturquoise* module negatively correlated to obesity traits.

The *skyblue* and *steelblue* modules were close to each other in the hierarchical clustering (Fig 2A) and they were both enriched for the GO component "extracellular region" (FDR = 6.4 10^-3^ and 1.8 10^-5^, respectively). These modules contained several genes coding for ECM constituents, in particular collagens, as well as proteins involved in cell adhesion, cell-cell interaction, peptidase activity and cytokine activity. The *skyblue* module was associated to a network of weakly interconnected hubgenes (Fig 5). The strongest correlation was between *PAMR1*—a gene reported to modulate phagocytosis, a process contributing to normal tissue remodeling [29], and *SCARA5*—a scavenger receptor with a collagen-like domain suspected to be involved in adipogenesis [30]. The *skyblue* module positively correlated to glucose (r = 0.45) and negatively to obesity-related traits.

By contrast, the *steelblue* module was associated to a network of strongly interconnected hubgenes (Fig 5) which exhibited a great stability. Among the most highly connected genes were *PPL* (periplakin)—a component of the cornified envelope, *PDGFRA*—the platelet-derived growth factor receptor, *DCN* (decorin)—a component of connective tissue, *C1R* and *C1S* —two subcomponents of the complement molecule C1, and *CTSK* (cathepsin K) which is involved in ECM remodeling and was strongly associated to *ECM1* (extracellular matrix protein 1). As the *skyblue* module, the *steelblue* module negatively correlated to obesity-related traits, especially BMI, adipocyte volume and leptin (r = -0.46, -0.48 and -0.51, respectively).

The *violet* module was enriched in the GO processes "lipid biosynthesis" (FDR = 6.3 10^-9^), "cholesterol metabolism" (FDR = 8.2 10^-6^) and "fatty acid metabolism" (FDR = 1.4 10^-5^). The top hubgenes were highly interconnected (Fig 5). In particular, strong correlations were observed between *INSIG1* that plays a key role in the control of cholesterol synthesis and *ACLY*, *FAD5* and *IDI1*, all involved in cholesterol and fatty acid synthesis. Another small highly interconnected sub-network was constituted of *ELOVL5*, *ME1* and *SPTSSA*, the 2 former being involved in fatty acid synthesis and the latter in sphingolipid synthesis. *SREBF1*, a transcription factor that binds to the sterol regulatory element-1 (SRE-1) and regulates lipid metabolism was also a top hubgene. Among the top 30 hubgenes, 16 were significantly downregulated at T3, a result consistent with the aforementioned enrichment of lipid pathways in differentially expressed genes. The *violet* module positively correlated to CRP (r = 0.49) and negatively, although not significantly, to lipid traits.

Finally, the *plum* module, despite a moderate stability (Fig 2B), deserved a special interest because of its strong enrichment for the GO process "response to virus" (FDR = 1.4 10^-4^). Almost all positive hubgenes of the network encoded proteins involved in the IFN-induced pathway and this sub-network was highly stable. *CRY1* encoding a circadian protein was negatively correlated to IFN-induced genes (Fig 4). The *plum* module positively correlated to adiponectin (r = 0.49).

Remarkably, the 3 most stable modules (*turquoise*, *magenta* and *tan*), to which could be added the IFN-related *plum* sub-module, were all reflecting innate and adaptive immune pathways. Because of the key role of immune cells in the low-grade inflammation associated to obesity [2], we focused further analyses on these modules.

### Modifications of immune response-related modules after surgery

**Fig 6:**
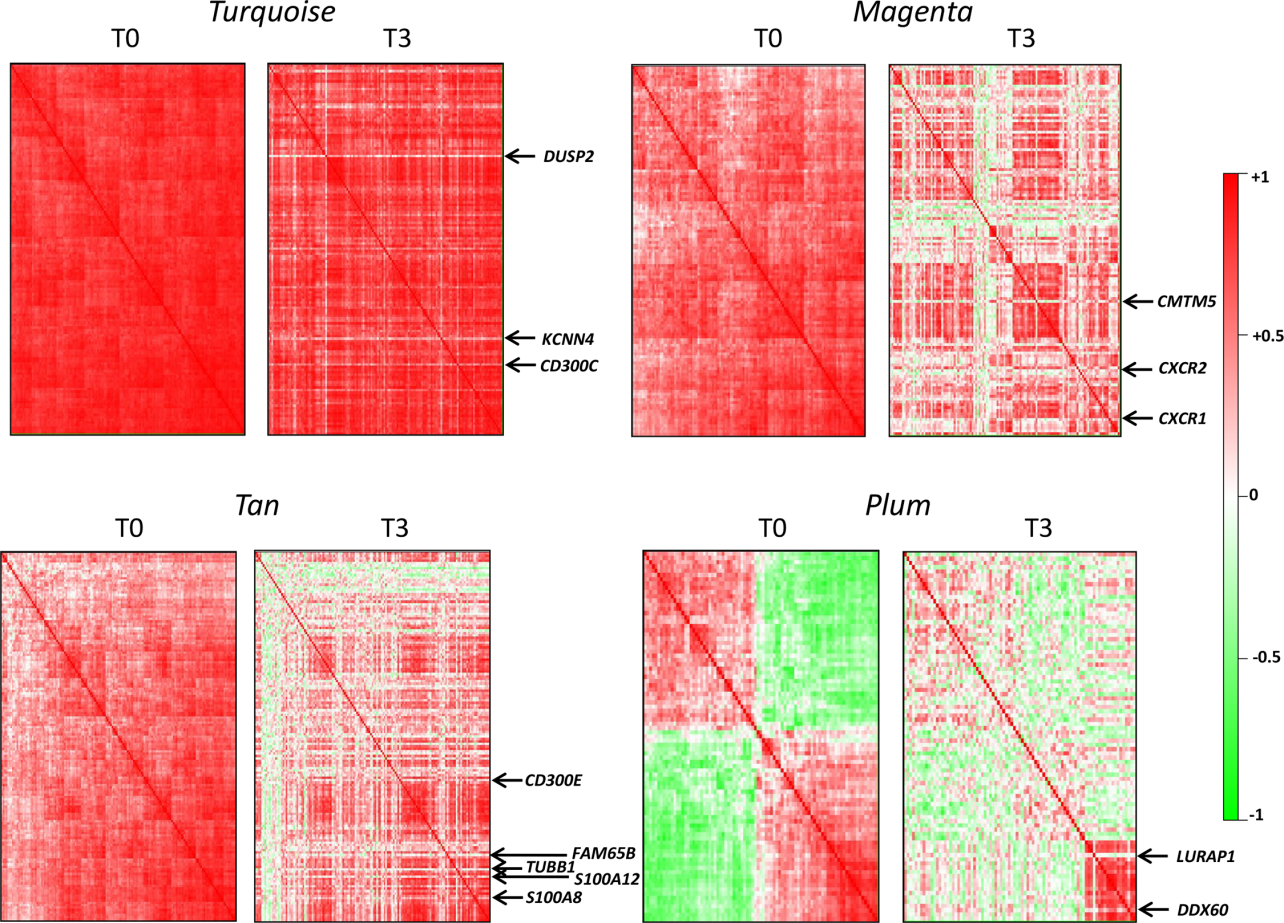
Modifications of immune-response networks after surgery-induced weight loss. Heatmaps representing pairwise correlation matrices of gene expressions are displayed for each module. At T3, genes are ranked in the same order as in T0. Only genes having the highest membership to each module are displayed, except for the *plum* module where all genes are shown.

We investigated whether the improvement of the inflammatory profile following surgery might be reflected by modifications of immune cell pathways. For this purpose, correlation heatmaps of immune response-related modules were compared between T0 and T3. For each module, we focused on genes having the highest contribution (module membership) to the module. A general view of the network changes is displayed in Fig 6 while detailed heatmaps at T3 are given in S1, S2, S3 and S4 Figs.

The *turquoise* module, reflecting a general immune response, conserved a highly dense connected network at T3, except a few genes losing association with the network (S1 Fig). The most striking one was *DUSP2* which encodes a member of the dual specificity protein phosphatase subfamily that regulates MAPK signaling and has a critical role in inflammatory response of macrophages [31]. Other genes disconnected from the module at T3 were *KCNN4* coding for a component of a calcium-activated potassium channel in T-lymphocytes, and *CD300C* coding for a member of the CD300 family of receptors that regulate immune cell processes [32].

The *magenta* module associated to lymphocyte expression showed profound perturbations after surgery (S2 Fig). The dense matrix of expressions at T0 was split into several disconnected subsets at T3. A first highly preserved sub-module comprised several hubgenes which characterized lymphocyte activation. The only gene disconnected from this block at T3 was *CMTM5*—a gene belonging to the chemokine-like factor gene superfamily with putative tumor suppressor activity. A second subset contained genes involved in signal transduction such as chemokine receptors (*CXCR1*, *CXCR2*, *CCR7)*, G-protein-coupled receptor (*GPR97*) and interleukin receptors (*IL7R*, *IL18RAP*). This group exhibited looser correlations at T3, especially *CXCR1* and *CXCR2* were no longer associated with other module members. Worthy of note, the membership of hemoglobin and erythrocyte-specific genes to the module at T0 disappeared at T3. This loss of correlation was accompanied by a marked decrease of hemoglobin gene expressions at T3. These observations may be explained by a higher blood cell contamination at baseline.

The *tan* module reflecting neutrophil expression also showed important modifications after surgery (S3 Fig). Although *S100A8* and *S100A9* were still coexpressed at T3, *S100A8* exhibited a lower pattern of correlations with other genes than *S100A9*, suggesting a dissociation of the dimer *S100A8*/*A9* involved in neuthrophil activation. Similarly *S00A12*—the 3^rd^ calgranulin gene—lost association with other module members. Several other hubgenes which were densely connected at T0 were no longer coexpressed at T3, such as *VNN2* encoding vanin 2—a protein involved in migration of neutrophils, *TUBB1* (tubulin β1 involved in cell migration), *FAM65B* which dampens chemokine-induced T cell migration [33], *APOBEC3A* which mediates antiviral immunity, and *FPR2* which encodes a receptor mediating activation of neutrophils.

A small *tan* sub-module highly conserved at T3 included several genes involved in the immune response mediated by dendritic cells (*CLEC7A*, *CLECL1*, *TLR1*, *TLR6*, *LY75*, *PIK3CG*, *SIGLEC10* and *LILRA5*). Interestingly, *CD300E*, another member of the CD300 receptors which, contrary to the ubiquitously leukocyte-expressed *CD300C*, is specifically expressed in monocytes and dentritic cells, completely lost its association with the sub-module (S3 Fig).

Within the *plum* module, the highly interconnected cluster of genes reflecting the IFN-signaling pathway was strongly preserved at T3. There were, however, two notable exceptions: *DDX60* and *LURAP1* (S4 Fig). *DDX60* positively regulates *DDX58* and *IFIH1*-dependent type I interferon response to viral infection [34]. *LURAP1* (previously *C1orf190*) [35] is an activator of the NF-κB signaling pathway which plays a crucial role in obesity-induced inflammation [36].

We next investigated to what extent the main genes cited above were also differentially expressed after surgery (Table 2). Almost all these genes showed a tendency towards downregulation at T3, and 9 of them exhibited a nominally significant decrease of expression after surgery (P<0.05).

**Table 2:** Differential expression by time of the main genes disconnected from immune-response modules after surgery.

Gene ID	Module	Time coefficient	Nominal P-value	FDR	Mean counts T0	Mean counts T3
*S100A8*	Neutrophils	-0.708	0.003	0.047	348	165
*CXCR1*	Lymphocytes	-0.678	0.005	0.058	33	18
*S100A12*	Neutrophils	-0.815	0.005	0.062	28	12
*FPR2*	Neutrophils	-0.849	0.007	0.069	17	7
*CD300E*	Neutrophils	-0.467	0.009	0.084	92	58
*FAM65B*	Neutrophils	-0.329	0.017	0.119	162	126
*CXCR2*	Lymphocytes	-0.385	0.025	0.149	76	54
*CCR7*	Lymphocytes	-0.264	0.034	0.179	82	69
*IL7R*	Lymphocytes	-0.442	0.044	0.209	44	29
*S100A9*	Neutrophils	-0.324	0.092	0.314	721	457
*APOBEC3A*	Neutrophils	-0.212	0.104	0.337	78	66
*DUSP2*	Macro/mono	-0.240	0.158	0.420	65	54
*VNN2*	Neutrophils	-0.198	0.167	0.431	58	46
*CMTM5*	Macro/mono	-0.173	0.232	0.509	11	10
*GPR97*	Lymphocytes	-0.186	0.432	0.690	16	13
*LURAP1*	Interferon	0.070	0.582	0.794	29	33
*DDX60*	Interferon	-0.024	0.621	0.817	549	536
*KCNN4*	Macro/mono	-0.083	0.627	0.821	142	143
*CD300C*	Macro/mono	0.071	0.688	0.857	67	72
*IL18RAP*	Lymphocytes	-0.049	0.735	0.880	41	43
*TUBB1*	Neutrophils	0.069	0.741	0.883	100	106

**Fig 7:**
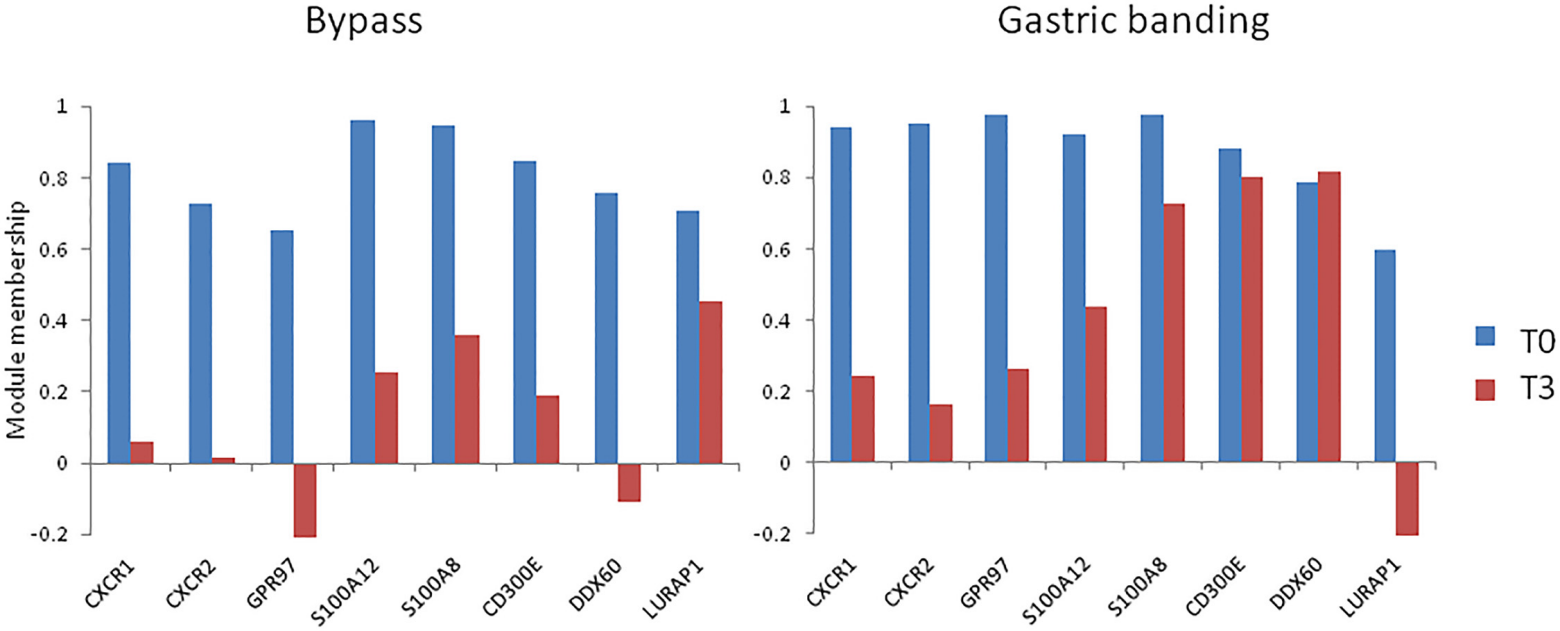
Module membership of some genes of interest before (T0) and after surgery (T3), separately in RYGB and AGB. The module membership of a gene represents the correlation to its module eigengene. For each module, the eigengene at T3 was calculated on the same gene set as at T0.

Lastly, we checked whether modifications were dependent of the type of surgical procedure. For this purpose, we compared the module membership of genes (i.e. the correlation with their module eigengene) at T0 and T3, separately in RYBG and AGB. This analysis showed that the decrease of correlation of *CXCR1*, *CXCR2*, *GPR97*, *S100A8* and *S100A12* to their module eigengene was very similar after RYBG and AGB (Fig 7).

### The IFN-signaling module in obese versus lean mice

**Fig 8:**
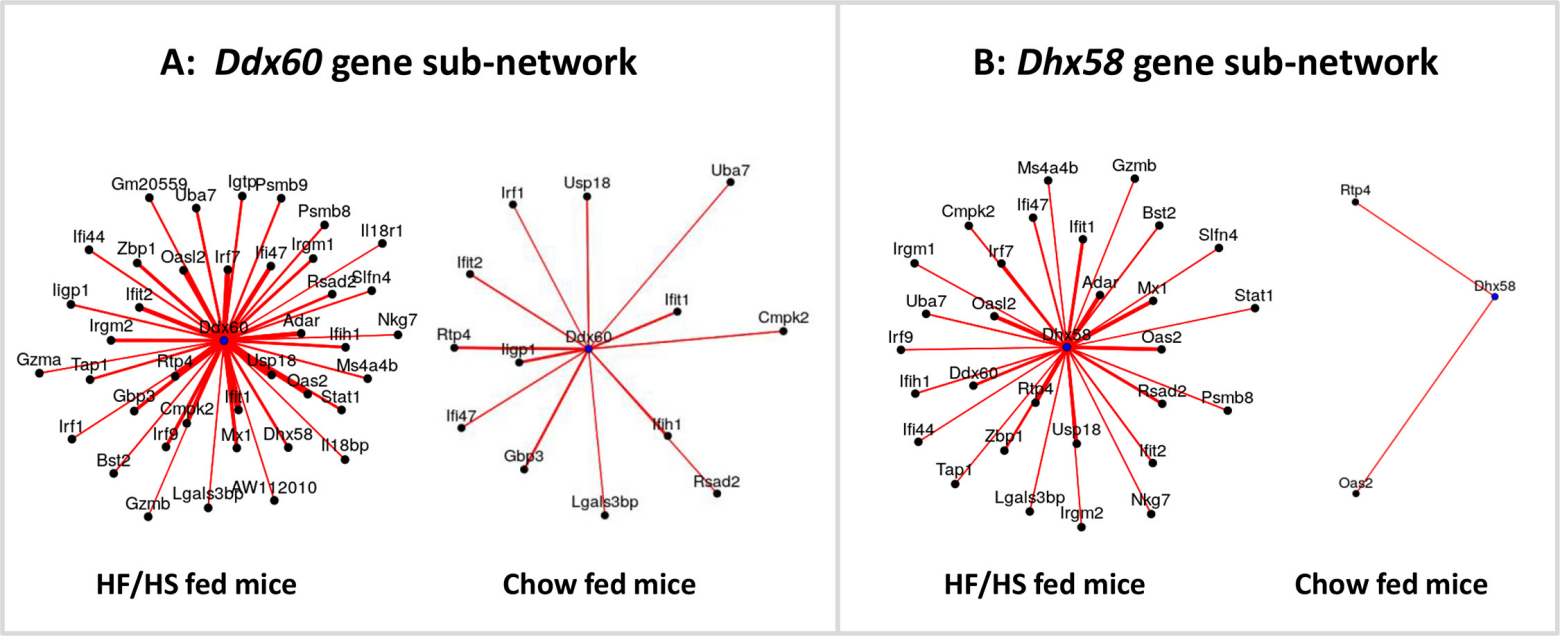
Sub-networks of expression around *Ddx60* and *Dhx58* genes in obese versus lean mice. Comparison of obese mice fed a high-fat/high-sucrose (HF/HS) diet and lean mice fed a chow diet (microarray data). Only edges corresponding to significant correlations are shown (P<0.05 and cor>0.4). The thicker and shorter edges correspond to stronger correlations. Networks were visualized using the gplot function of the sna R package.

For sake of validation, we applied WGCNA to transcriptomic data of adipose tissue from obese mice fed a high fat/high sucrose (HF/HS) diet. Among 9 detected modules, one module including 63 genes was highly enriched for genes of the IFN-signaling pathway (P = 1.6 10^-6^). In particular, this module included 9 genes common to the human *plum* module: *Cmpk2*, *Ddx60*, *Ifi44*, *Ifih1*, *Ifit1*, *Irf7*, *Mx1*, *Oas2* and *Rsad2*. This module showed a high preservation in lean mouse fed a chow-diet (S5 Fig). However, similar to what was observed in humans after bariatric surgery, most of the connections of *Ddx60* in the obese state were lost in the lean state (Fig 8A). A similar pattern was observed for *Dhx58*, another regulator of the *DDX58* and *IFIH1*-mediated signaling cascades (Fig 8B).

## Discussion

In the present study, the impact of bariatric surgery on SAT transcriptome was studied by two approaches: first by measuring the modifications in single gene expressions after surgery, and second by investigating the perturbations in networks of coexpressed genes. The first approach informs about the difference in the abundance of a given molecule, while the second approach provides insight into mechanisms of rewiring of transcriptional networks in response to surgery-induced weight loss. While the two approaches are complementary, the latter one has the advantage of providing a more informative picture of the dynamic changes in the gene regulatory networks [37].

Using a stringent 5% FDR, we identified 1214 genes whose expression significantly varied between T0 and T3. Upregulated genes were mostly involved in the basal cellular machinery and reflected increased protein synthesis and decreased degradation as already reported [8, 11]. This enhanced biogenesis is probably a requirement of the organism for counteracting the caloric restriction caused by the surgical procedure. The upregulation of the IFN-γ induced pathway may be explained by the fact that immunoproteins are thought to be highly demanding in terms of energy and may be particularly affected by energy restriction [38]. As already known from microarray studies [7, 8, 11], downregulated genes were enriched in major metabolic functions of adipose tissue such as ECM organization and metabolism of lipids, fatty acids and carbohydrates. The enrichment of downregulated genes in the KEGG category "Packaging telomere ends" is of particular interest given the inverse link reported between adiposity and telomere length [39].

By WGCNA, we identified 26 modules of coexpressed genes at baseline, 8 of them showing a great stability by Jackknife analysis. Worthy of note, modules having the largest proportion of DE genes were among those having the lowest stability. This finding may be explained by the fact that DE genes are genes that are less constrained in terms of variability than those that are tightly regulated within systems sustaining homeostasis of the organism. In keeping with this interpretation, we have previously shown that gene expressions that are under the influence of genetic variants are more likely to be also modulated by non-genetic factors [40].

The 3 most stable modules at T0 (*turquoise*, *magenta* and *tan*) were related to the immune response. Immune modules are thought to reflect the low-grade chronic inflammation associated to obesity. This immune signature shared by many common chronic diseases and observed across several tissues has been shown to be driven by a few key genes [41]. Worthy of note, all the predicted key drivers of the "inflammatome" regulatory network described in human adipose tissue [41] were among the major contributors of the *turquoise* module.

The 3 immune response-related modules reflected the diversity of immune cells involved in adipose tissue inflammation. The *turquoise* module was a large module reflecting a non-specific signature of innate immune cells, mainly macrophages which are the most abundant immune cells in adipose tissue, but also monocytes. The *magenta* module reflected the adaptive immune response elicited by T lymphocytes which have an essential role in the propagation of adipose inflammation [19]. The *tan* module characterized the activity of neutrophils which play a primary role in the initiation of inflammation by recruiting macrophages, dendritic cells and lymphocytes to fat depots [3].

Three months after surgery, the 3 immune response-related modules exhibited distinct patterns of modifications. The *turquoise* module was strongly preserved with very few exceptions of disconnected genes at T3. Among them, *DUPS2* is of particular interest for its potential as a therapeutic target to modulate immune effectors functions [31]. The dissociation of *CD300C* from the *turquoise* module, parallel to that of *CD300E* from the *tan* module, is also worthy of note knowing that CD300 molecules are important modulators of the immune system [32].

In the *magenta* module, the most striking feature was the disconnection between genes involved in T-cell signaling and receptors mediating the signal transduction such as *CXCR1*, *CXCR2*, *CCR7*, *IL7R* and *GPR97*.

In the *tan* module, the major modification induced by surgery was a downregulation of *S100A8* and *S100A12* and their dissociation from other genes of the module. These proteins are secreted by neutrophils upon activation of the immune system and participate in neutrophil-mediated inflammation [21]. Several other genes losing their module correlation were involved in neutrophil chemotaxis and migration (*FPR2*, *VNN2*, *TUBB1*, *FAM65B*). These results suggest a critical role of these factors in dampening the migration and the activity of neutrophils in adipose tissue after surgery.

Interestingly, several of these genes (*S100A8*, *S100A12*, *CXCR1*, *CXCR2*, *CCR7*, *IL7R*, *FPR2*, *CD300E* and *FAM65B*), besides being disconnected from their module after surgery, were also significantly downregulated, suggesting that the encoded molecules—mostly receptors and ligands—play a key role in the rewiring of inflammatory transcriptional networks after surgery-induced weight loss.

The present study also highlighted a small network of strongly interconnected genes which comprised 19 IFN-related genes. A similar module was identified in circulating monocytes of the same obese subjects (data not shown) and was also present in mice fed an obesogenic diet. One member of the network was *IRF7*, a master regulator of type I IFN-dependent immune response [42]. *IRF7* has been identified as the key driver of an inflammatory network associated to type 1 diabetes [43], which greatly overlaps with the IFN network found in the present study. *IRF7* has also been shown to contribute to diet-induced obesity and insulin resistance [44]. Interestingly, the strongest negative contributor of the *plum* module—reproducibly found in almost all sub-analyses—was *CRY1*, a circadian gene. The negative association of *CRY1* with IFN-induced genes is consistent with the protective role of *CRY1* in the modulation of inflammation by the circadian clock [45]. A striking finding was the dissociation of *DDX60* from several IFN-induced genes after surgery. A similar result was observed when lean mice were compared to obese mice. *DDX60* has been recently described as a novel antiviral factor involved in RIG-I-mediated IFN signaling [34]. The present finding suggests that *DDX60* may represent a potential therapeutic target for attenuating obesity-associated inflammation.

Several limitations of the present study should be acknowledged. First, it was based on a limited sample size, even though this is the largest study, to our knowledge, using the RNA-seq technology for assessing the transcriptome of human adipose tissue. Second, the visceral fat compartment is known to become more pro-inflammatory during obesity that the subcutaneous fat compartment. However, visceral fat sampling needs an invasive surgical biopsy and therefore could not be obtained in the post-surgical follow-up. Third, adipose tissue represents a heterogeneous mixture of cells composed of adipocytes and stromal vascular cells, and the cell distribution may differ across individuals and before and after surgery. The lack of tissue samples for immunohistochemical or cytometry analyses did not allow us to quantify the abundance of the different types of immune cells. Fourth, adipose tissue remodeling after bariatric surgery is a very dynamic process, and analyzing the expression profile at a single postsurgical time point only represents a snapshot that may not illustrate the full picture of adipose remodeling. Fifth, to keep gender homogeneity, the study was restricted to women who represent the vast majority of patients undergoing bariatric surgery, but this may have introduced a source of variability induced by menstrual cycle variations and/or altered sex hormones on fat biology. Finally, functional experiments targeting the genes in the pathways highlighted by the present study are needed to firmly establish their role in the modulation of inflammation after bariatric surgery.

## Methods

### Subjects

The study population initially included 24 obese women undergoing bariatric surgery recruited at Pitié-Salpêtrière Hospital (Nutrition Department, Institute of Cardiometabolism and Nutrition, ICAN). Following usual guidelines, all patients met the criteria for needing obesity surgery: these included a BMI ≥40 kg/m² or ≥35 kg/m² with at least one comorbidity (hypertension, T2D, dyslipidemia or obstructive sleep-apnea syndrome). Preoperative evaluation included a detailed medical history, a physical examination, and assessment of nutritional, metabolic, cardiopulmonary, vascular and psychological factors. The weight of the subjects had been stable (variation < 2 kg) for at least 3 months prior to surgery. Subjects did not demonstrate evidence of acute or chronic inflammatory disease, infectious diseases, cancer and/or known alcohol consumption (> 20g per day) at any time point. Clinical and biological parameters were assessed prior to surgery (T0) and at 3 months after surgery (T3).

### Ethics statement

All subjects gave their written informed consent to participate in the study. The Ethics Committee of the “*Comité de Protection des Personnes Ile de France 1*” (Hôtel-Dieu Hospital) approved the clinical investigation. The study was conducted in accordance with the Helsinki Declaration and was registered in a public trials registry: ID Number NCT01454232.

Fat mass was measured by whole-body fan-beam dual energy x-ray absorptiometry (DXA, Hologic Discovery W, software v12.6; Hologic, Bedford, MA). Subjects were considered to have diabetes if they were taking an antidiabetic treatment or if fasting glycemia was ≥7 mmol/l on at least two different occasions. To estimate the patients cardiometabolic risk, we used the thresholds established by the International Diabetes Federation (IDF, 2005) for the definition of MetS. Subjects were included in the MetS group if they had at least two of the four factors: 1) raised triglyceride levels (≥ 1.7 mmol/L) or specific treatment; 2) reduced HDL-cholesterol < 1.29 mmol/L) or specific treatment; 3) raised blood pressure (systolic ≥ 130 or diastolic ≥ 85 mmHg) or treatment of previously diagnosed hypertension; 4) raised fasting plasma glucose (≥ 5.6 mmol/L) or previously diagnosed T2D.

SAT samples were obtained by needle biopsy from the peri-umbilical area under local anesthesia (1% xylocaïne) and venous blood samples were collected in the morning after a 12-hour fast for routine determination of blood cell counts, glucose, insulin, triglycerides, total cholesterol, LDL-cholesterol, HDL-cholesterol, leptin, adiponectin and IL-6 as previously described [46]. Insulin resistance was assessed using the HOMA-IR index: insulinemia (mUI/L) × fasting blood glucose (mmol/L)/22.5. High sensitive CRP was measured using an immunoturbidimetric assay on a Cobas 501 analyser (Roche Diagnostics, Meylan, France).

### mRNA preparation and sequencing

Total RNA was extracted from SAT using the *mir*Vana miRNA Isolation Kit (Ambion). 500 ng of total RNA were used to prepare the library with TruSeq RNA sample Prep Kit V2 (*Illumina*). Briefly, mRNA was selected from total RNA by poly-T oligo-attached magnetic beads and fragmented. Reverse transcription was done with random hexamers by SuperScript II reverse transcriptase. From resulting cDNA, classical library preparation steps included end repair, adenylate 3 ends, adaptors ligation with specific index and 15 cycle-enrichment amplification performed according to manufacturing protocol. The generated libraries were submitted to quality control on High Sensitivity DNA chip from the *Agilent* Bioanalyzer 2100 (Agilent Technologies, Wokingham, UK) and quantified by fluorimetry using the Quant-iT dsDNA Assay Kit (*Life Technology*). A dilution at 10nM was performed for each library and pools of 12 libraries were constituted. Each pool included the libraries from 3 individuals for the 2 time points, each library being identifiable by a specific index. The resulting pools were loaded on a High Sensitivity DNA chip (*Agilent*) and quantified by integration. 9 pM of this solution was used for cluster generation using the TruSeq PE Cluster Kit v3 (*Illumina*) on a CBot instrument. A 75 bp paired-end run was performed on the *Illumina* HiSeq2000 platform with the TruSeq SBS kit V3 (*Illumina*). Sequencing was performed on two 8-laned flow cells. Each flow cell included 4 pools sequenced twice in 2 different lanes. The two flow cells were processed in the same conditions at 2 months of interval (runs 1 and 2, respectively).

### RNA-seq data pre-processing

Reads of bad quality were filtered out using fastq_illumina_filter. Trimming was performed using sickle with default parameters (quality threshold = 20, length threshold = 20). Orphan reads were excluded. Quality of reads was checked using FastQC. Paired-end reads were mapped to the human transcriptome using Bowtie2 v2.1.0 (http://bowtie-bio.sourceforge.net/bowtie2/). The reference human transcriptome was generated from the UCSC reference genome (hg19) and the *Illumina* iGenome annotation file of transcripts using RSEM [47]. Quantification of transcript abundance was performed using eXpress [48]. The data used for analysis were the eff_counts which give the estimated number of fragments mapping to a given target sequence adjusted for fragment and length biases [49]. Analyses were performed at the gene level by summing isoform counts for each gene.

### Statistical analysis

After exclusion of 2 individuals failing to be sequenced because of bad quality or insufficient amount of RNA, analysis was performed in 22 subjects in the R environment (v3.0.2). Gene coverage was assessed using the RSeQC package (http://rseqc.sourceforge.net/). The plot showed a well-documented 3' bias due to polyA selection, but there was no clear relationship between the skewness of the coverage and the RNA quality assessed by RIN values (S9 Table, S6 Fig). We further checked by principal component analysis whether samples with low RIN may behave as outliers, but no outlier was detected. Genes with low expression levels, defined by a mean number of counts < 10 at both times, were filtered out. We also excluded a few genes annotated as micro RNAs (MIR symbol), leaving out 15,972 genes for analysis.

#### Statistical model for differential analysis by time

For each gene, the difference of expression level between T0 and T3 was estimated using a negative binomial regression model fitted on read counts. We used the R hglm package [50] which allows fitting fixed and correlated random effects resulting from repeated time measures. Counts were normalized using the size factor estimated by the R DESeq package [51]. This factor accounts for variations of library size among samples and corrects for the bias induced by highly expressed genes. It was introduced as an offset in the negative binomial model. Fixed effects were time (T3 vs T0), age, ethnicity (European vs non European), run, RIN and RNA concentration. Additionally we tested whether time effects differed according to the type of surgical procedure by introducing the surgical procedure (RYGB vs AGB) and an interaction term time*procedure in the model. Parameters and their standard errors were estimated by the h-likelihood and tested by means of the t-test. To account for multiple testing, we used the FDR [52] and considered as significant an FDR < 0.05.

#### Gene Set Enrichment Analysis (GSEA)

The GSEA software (v2.0.12) was used to investigate whether the most differentially expressed (DE) genes between T0 and T3 shared common biological functions [15]. The analysis was performed on the list of all genes ranked either by their signed or by their absolute t-statistics obtained from the negative binomial model. Parameters were set to their default value. Gene sets tested for enrichment were taken from the MSigDB database (BioCarta, KEGG and REACTOME gene sets). The statistical significance of the enrichment score was assessed by performing 1000 permutations. Overlap among gene sets was examined by a leading edge analysis *(* www.broadinstitute.org/gsea/). We considered as significant a FDR < 0.05.

#### Weighted Gene Coexpression Network Analysis (WGCNA)

The R WGCNA package (v1.34) was used to identify networks of coexpressed genes at T0 [18]. Prior to analysis, data were transformed using the VST transformation proposed by the DESeq package. The resulting variable was further adjusted for run, RIN and RNA concentration and the 10,000 most variable gene expressions at T0 were used for analysis. We used the adjacency matrix to specify the connection strength *a*_*ij*_ between genes *i* and *j* (*a*_*ij*_ = |cor(*x*_*i*_,*x*_*j*_)|^*β*^). The power β was fixed to 6 after applying the function pickSoftThreshold. The topological overlap measure was used to define modules of interconnected genes.

Modules were merged using the Dynamic Tree Cut function [53]. Parameters were fixed to their default values. Spearman's rank correlation coefficients between module eigengenes and phenotypic traits were represented by a correlation heatmap. The module membership (MM) measured the correlation of a given gene expression profile with its module eigengene, genes with the highest rank of MM having the strongest contribution to the module.

Enrichment analysis of modules in functional GO categories was performed using the DAVID software (v6.7) [54] with the list of the 15,972 genes expressed in SAT taken as reference. A FDR < 0.05 was considered significant. For each module, the network of the 30 genes having the highest absolute MM in the module, thereafter named hubgenes [18], was displayed using the R function circlePlot.

Stability of modules was assessed by the Jackknife technique. The WGCNA algorithm was repeatedly run on the 22 subsets obtained by systematically leaving out 1 individual from the dataset and in each sub-analysis, we selected the module having the maximum overlap with each original module by means of the Jaccard index [55]. The mean and standard deviation of the maximum Jaccard indices were calculated over the 22 subsets of *n*-1 individuals. In order to select the most stable modules, for each gene *i* we computed the number *k*_*i*_ of times it was correctly classified in the best matching module of the different sub-analyses. We considered that a gene was steadily clustered if it was attributed to the right original module in ≥ 18 of the 22 sub-analyses. We repeated the same procedure by using the correlation between eigengenes instead of the Jaccard index, i.e. we selected the module having the maximum eigengene correlation with the original module.

### Microarray data in obese versus lean mice

These data have been previously described [56]. Briefly, mice from the Hybrid Mouse Diversity Panel including more than 100 inbred mouse strains were purchased from the Jackson Laboratory and bred in control (chow diet) and “obese” conditions at University of California, Los Angeles. Male mice of the obese group were maintained on a chow diet (Ralston Purina Company) until 8 weeks of age when they were given a high-fat/high-sucrose (HF/HS) diet (Research Diets-D12266B) with the following composition: 16.8% kcal protein, 51.4% kcal carbohydrate, 31.8% kcal fat. Age-matched control male mice were fed a chow diet (18% kcal fat) during 16 weeks. Total RNA was extracted from flash frozen epididymal adipose samples using Qiazol and the Adipose Tissue RNAeasy kit (Qiagen) according to the manufacturer's instructions. Gene expression levels were assessed using *Affymetrix* Mouse Genome HT_MG‐430A arrays and data were processed as previously described [57]. To investigate the preservation of gene expression modules between HF/HS and chow diet-fed mice, we applied the same pipeline of analysis as in humans. Modules were first detected in the HF/HS group by WGCNA and the within-module correlations were then compared to those in the chow diet group.

## Supporting Information

S1 Fig Modifications of the *turquoise* network after surgery-induced weight loss. The heatmap representing the pairwise correlation matrix of gene expressions is shown. Only genes having the highest membership to each module are displayed. For purpose of comparison, genes are ranked in the same order as at T0.
(PDF)

S2 Fig Modifications of the *magenta* network after surgery-induced weight loss. See legend of S1 Fig.
(PDF)

S3 Fig Modifications of the *tan* network after surgery-induced weight loss. See legend of S1 Fig.
(PDF)

S4 Fig Modifications of the *plum* network after surgery-induced weight loss. See legend of S1 Fig.
(PDF)

S5 Fig Heatmaps of the interferon-related module in obese versus lean mice. Obese mice were fed a high-fat/high-sucrose (HF/HS) diet and lean mice were fed a chow diet. Heatmaps represent the pairwise correlation matrix of gene expressions assessed by microarray.
(PDF)

S6 Fig Plot of gene coverage of the 44 samples (22 individuals at 2 times). (A) Reads coverage according to position in the gene from 5' to 3'; (B) RIN values of the samples ranked by increasing 3' skewness of coverage.
(TIF)

S1 Table Comparison of expression levels between T0 and T3 for the 15,972 genes expressed in the subcutaneous adipose tissue. (XLSX)

S2 Table Parameter estimates from the full negative binomial regression including age, ethnic origin, run, RIN, RNA concentration, type of surgical procedure, time and time*procedure. (XLSX)

S3 Table Lists of upregulated genes contributing to enrichment in KEGG or REACTOME pathways by GSEA. The first sheet shows the GSEA report and each following sheet corresponds to an enriched pathway.
(XLSX)

S4 Table Lists of downregulated genes contributing to enrichment in KEGG or REACTOME pathways by GSEA. The first sheet shows the GSEA report and each following sheet corresponds to an enriched pathway.
(XLSX)

S5 Table Lists of genes contributing to enrichment in KEGG or REACTOME pathways in the GSEA analysis combining up- and downregulated genes. The first sheet shows the GSEA report and each following sheet corresponds to an enriched pathway.
(XLSX)

S6 Table Description of modules identified by WGCNA at baseline. (XLSX)

S7 Table Stability of modules identified at baseline assessed by the Jackknife technique leaving out 1 individual at each time. (XLSX)

S8 Table List of genes in the most stable modules. Each sheet of the Excel file corresponds to a different module.
(XLSX)

S9 Table RIN values of SAT samples at T0 and T3. (XLSX)
