# Supplementary figures and images for: Bariatric Surgery Induces Disruption in Inflammatory Signaling Pathways Mediated by Immune Cells in Adipose Tissue: A RNA-Seq Study
Source: PLoS One. 2015 May 4;10(5):e0125718. doi: 10.1371/journal.pone.0125718 (PMC4418598; doi:10.1371/journal.pone.0125718)

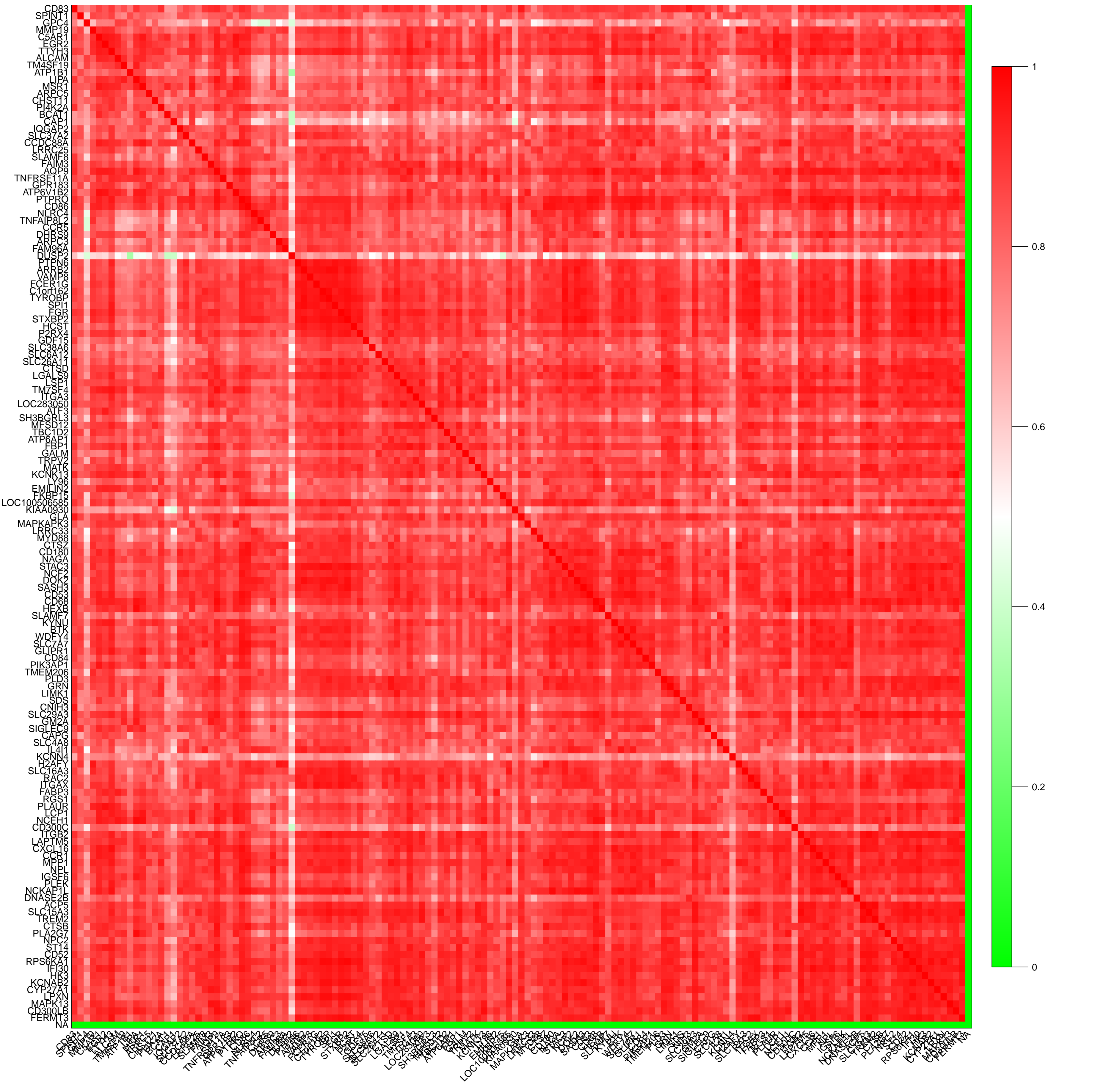

Supplement: S1 Fig — The heatmap representing the pairwise correlation matrix of gene expressions is shown. Only genes having the highest membership to each module are displayed. For purpose of comparison, genes are ranked in the same order as at T0. (PDF) [file pone.0125718.s001.pdf]

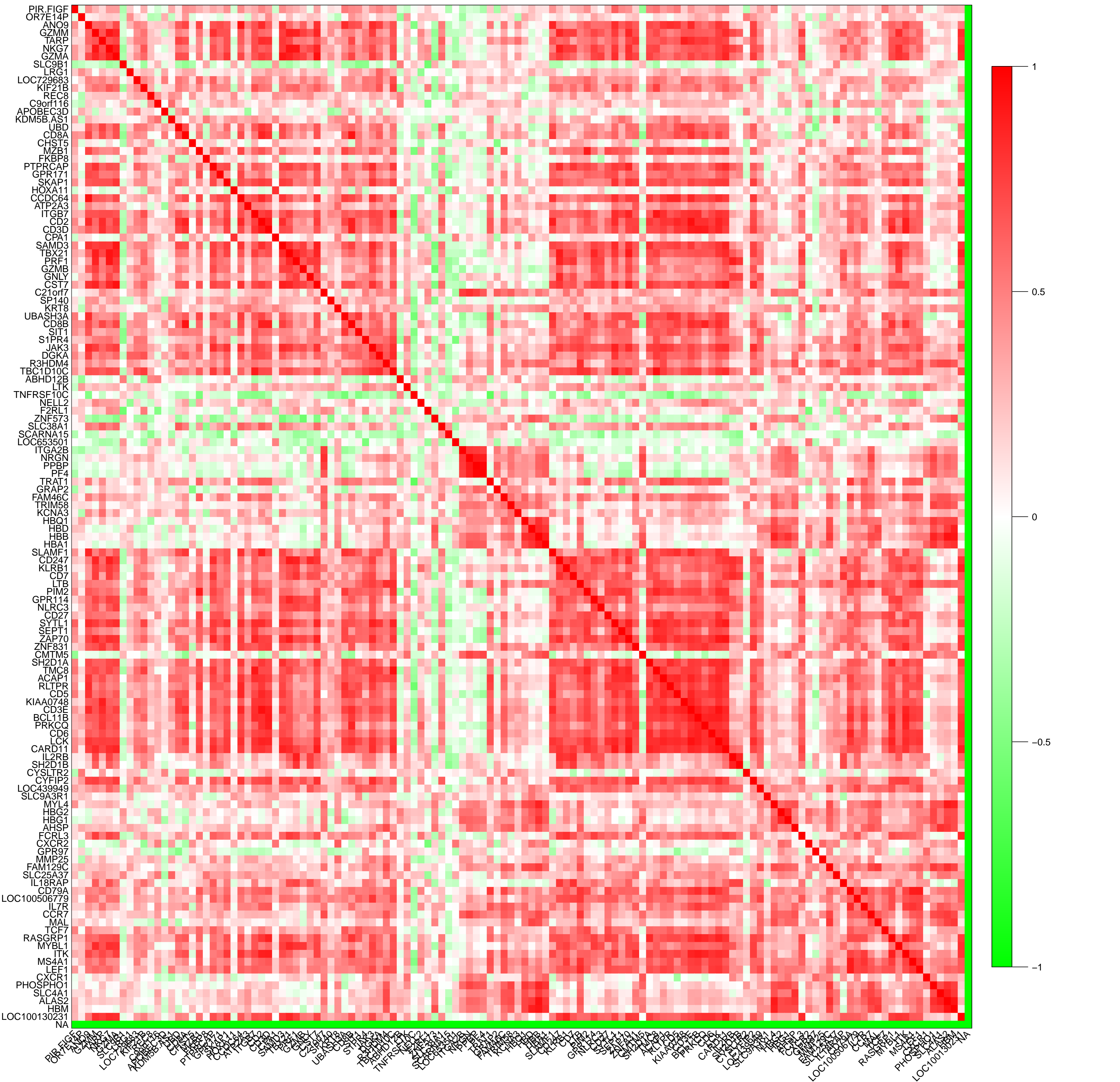

Supplement: S2 Fig — See legend of S1 Fig. (PDF) [file pone.0125718.s002.pdf]

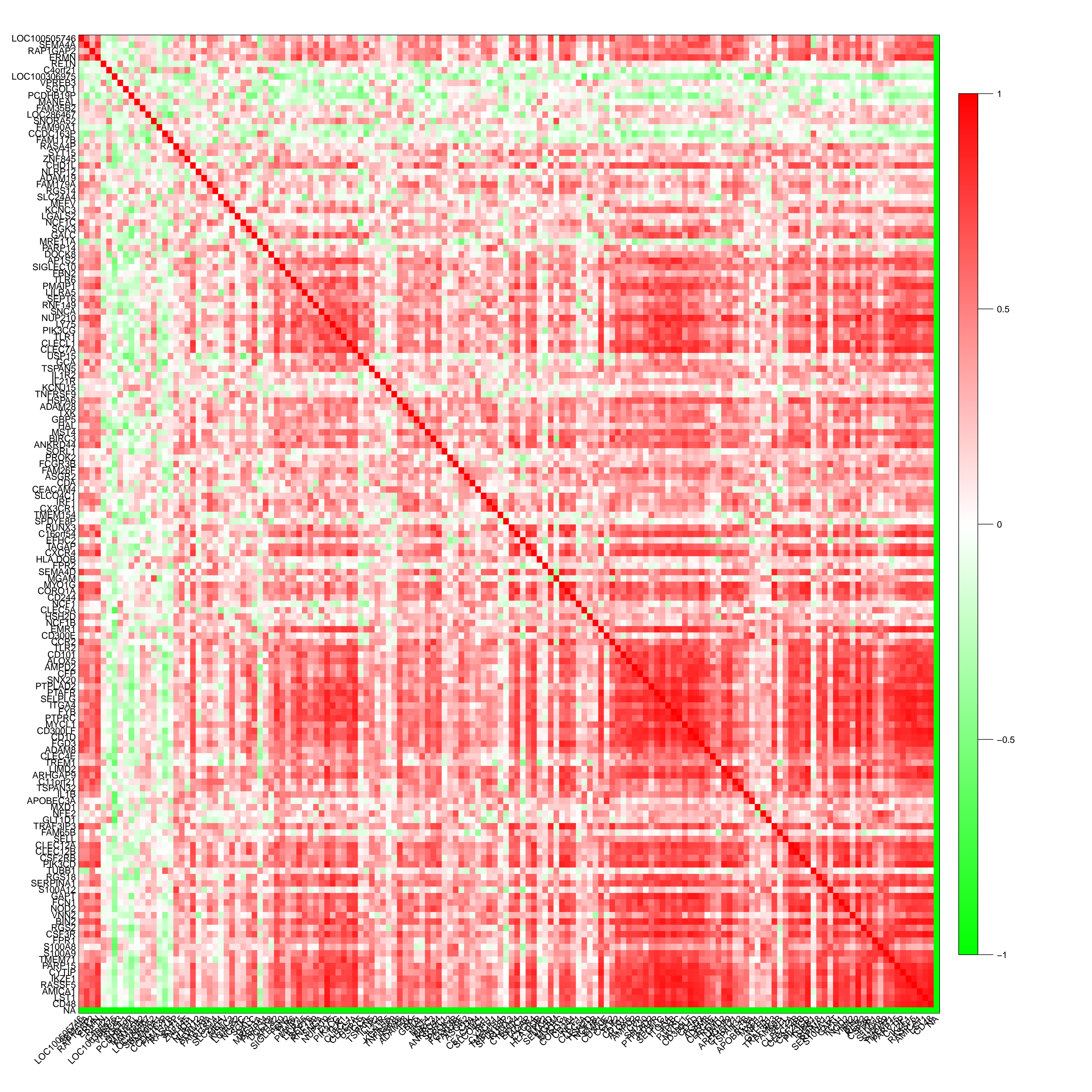

Supplement: S3 Fig — See legend of S1 Fig. (PDF) [file pone.0125718.s003.pdf]

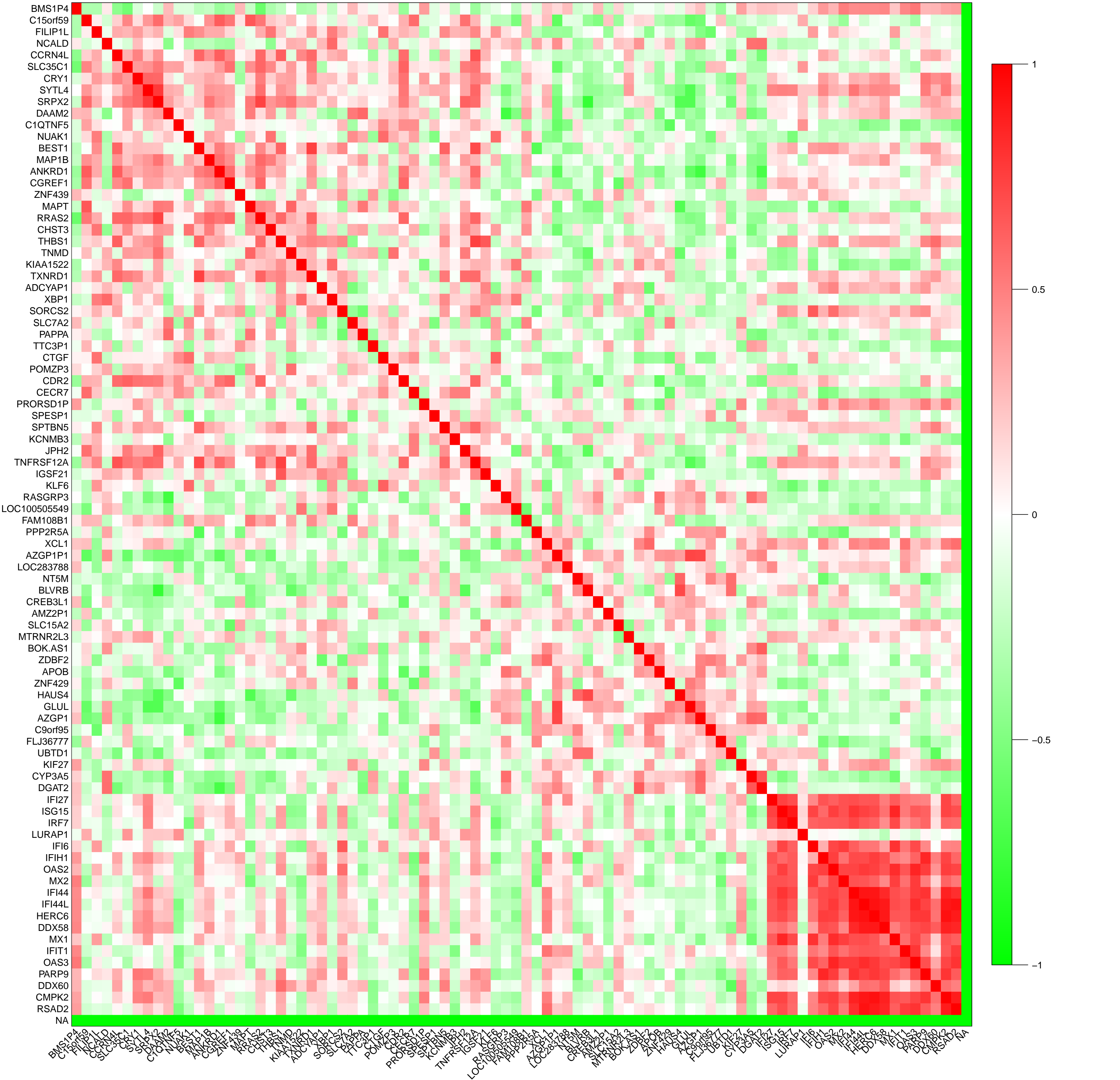

Supplement: S4 Fig — See legend of S1 Fig. (PDF) [file pone.0125718.s004.pdf]

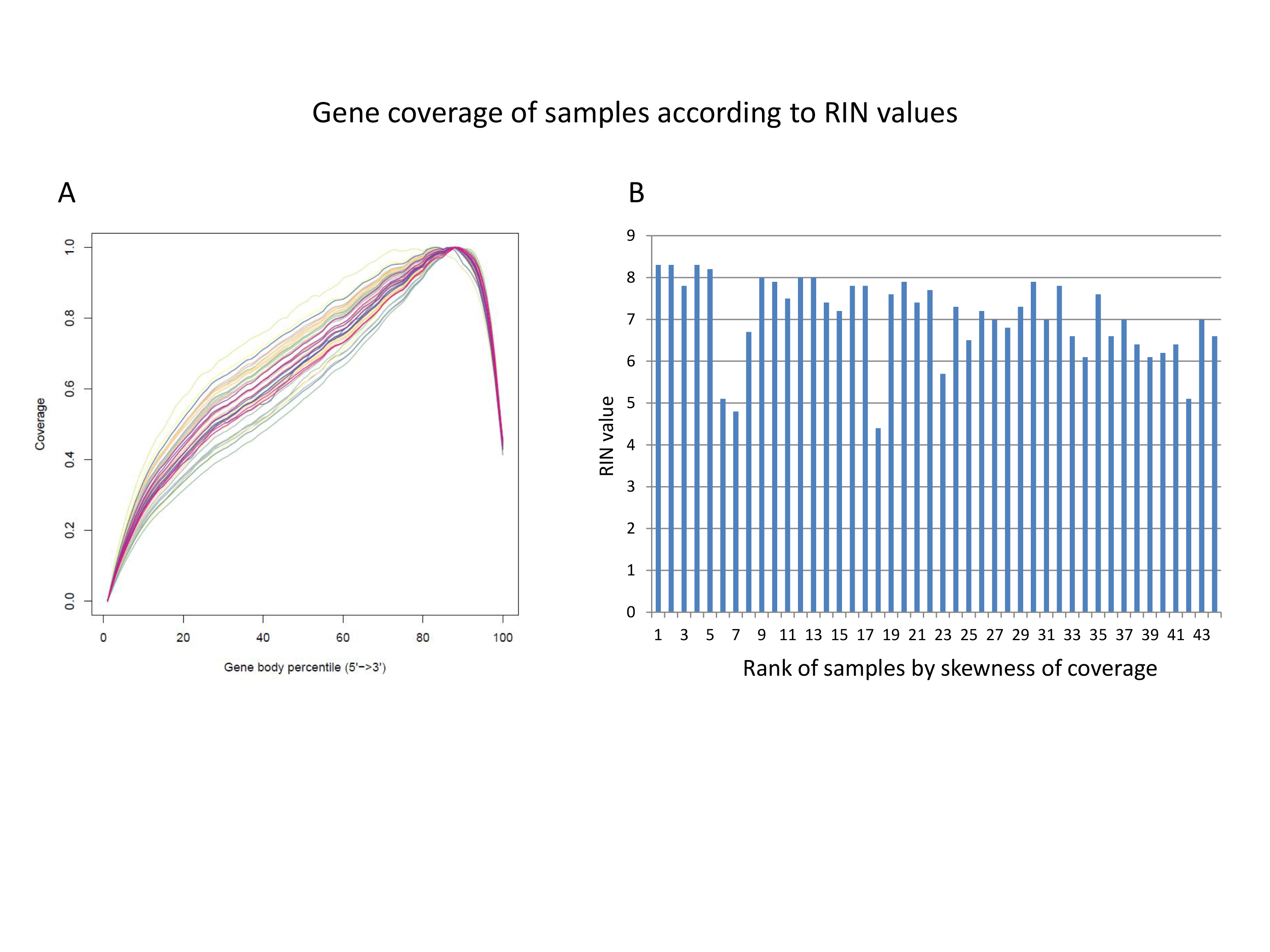

Supplement: S6 Fig — (A) Reads coverage according to position in the gene from 5' to 3'; (B) RIN values of the samples ranked by increasing 3' skewness of coverage. (TIF) [file pone.0125718.s006.tif]
