# Supplementary material for: Bariatric Surgery Induces Disruption in Inflammatory Signaling Pathways Mediated by Immune Cells in Adipose Tissue: A RNA-Seq Study
Source: PLoS One. 2015 May 4;10(5):e0125718. doi: 10.1371/journal.pone.0125718 (PMC4418598; doi:10.1371/journal.pone.0125718)
Supplement: S5 Fig — Obese mice were fed a high-fat/high-sucrose (HF/HS) diet and lean mice were fed a chow diet. Heatmaps represent the pairwise correlation matrix of gene expressions assessed by microarray. (PDF) [file pone.0125718.s005.pdf]

Heatmap of the “interferon” module in mice

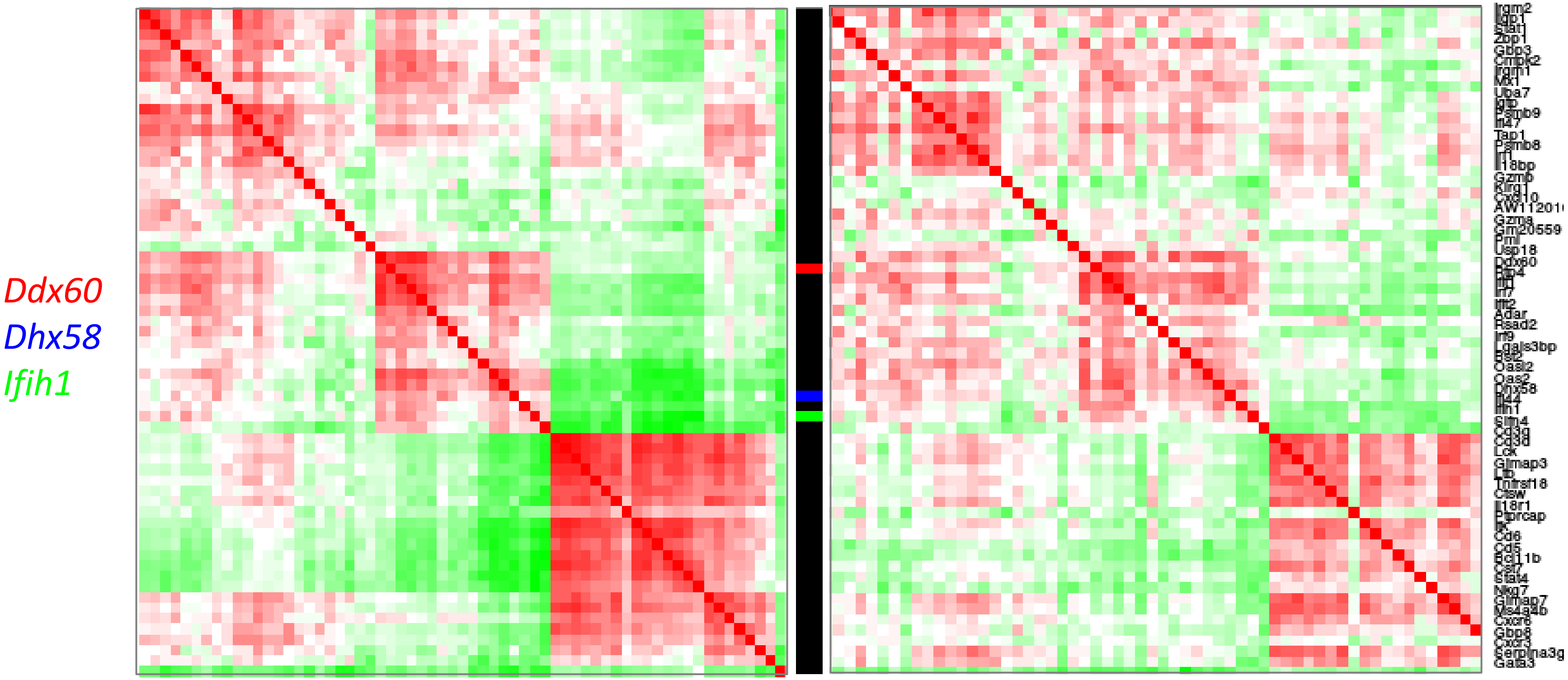

HF/HS fed mice

Chow fed mice
